# Effects of Pomegranate on Wound Healing

**DOI:** 10.7759/cureus.89032

**Published:** 2025-07-30

**Authors:** Ilkay Halicioglu, Nilgün Isiksacan, Firat Baytekin, Mehmet Kulus, Emre Gulbagci, Öznur Inan, Mehmet E Gunes

**Affiliations:** 1 Gastrointestinal Surgery, University of Health Sciences, Gazi Yasargil Training and Research Hospital, Diyarbakir, TUR; 2 Biochemistry, University of Health Sciences, Bakırköy Dr. Sadi Konuk Training and Research Hospital, Istanbul, TUR; 3 Pathology, University of Health Sciences, Çam and Sakura City Hospital, Istanbul, TUR; 4 General Surgery, University of Health Sciences, Çam and Sakura City Hospital, Istanbul, TUR; 5 Pathology, Edremit State Hospital, Balikesir, TUR; 6 Veterinary Medicine, SBU Istanbul Mehmet Akif Ersoy Thoracic and Cardiovascular Surgery Training and Research Hospital, Istanbul, TUR; 7 General Surgery, Faculty of Health Sciences, Istanbul Esenyurt University, Istanbul, TUR

**Keywords:** cecal ligation and puncture, pomegranate, rat model, sepsis, wound healing

## Abstract

Background: Sepsis disrupts normal wound healing and causes organ damage through a systemic inflammatory response. Pomegranate (*Punica granatum*) is a natural antioxidant rich in polyphenols, such as ellagitannins and punicalagins, and has demonstrated potential anti-inflammatory and tissue-healing properties in various preclinical models.

Objective: This study aimed to evaluate the effects of pomegranate extract administered before and after sepsis induction on wound healing in a cecal ligation and puncture (CLP) rat model.

Methods: Thirty-eight male Wistar rats were divided into four groups: sham (n = 8), sepsis (n = 10), pre-sepsis pomegranate (n = 10), and post-sepsis pomegranate (n = 10). Sepsis was induced via CLP. Pomegranate extract (100 µL) was administered by oral gavage for seven days before or after sepsis. Rats were sacrificed on postoperative day 7. Biochemical (alanine transaminase (ALT), AST (aspartate transaminase), urea, creatinine) and histopathological (granulation, lymphocyte infiltration, fibrosis, acute inflammation) parameters were evaluated. Statistical analysis included Kruskal-Wallis and Fisher's exact tests.

Results: ALT levels were significantly lower in the pre-sepsis pomegranate group compared to the sepsis (p = 0.003) and post-sepsis pomegranate (p = 0.009) groups. Creatinine levels were significantly lower in the post-sepsis pomegranate group compared to the sepsis group (p = 0.007). Granulation tissue scores were significantly lower in the pre-sepsis pomegranate group compared to the sepsis and post-sepsis pomegranate groups (p = 0.038). There was no significant difference in fibrosis or lymphocyte density between the groups.

Conclusion: Pretreating with pomegranate extract before inducing sepsis may protect against liver injury and support wound healing. Although the animals in this group were sacrificed earlier due to clinical deterioration likely related to the severity or timing of the sepsis, their biochemical and histological findings were favorable compared to those in the sepsis-only group. Our findings indicate that pomegranate extract, a readily accessible and well-tolerated natural product, may offer therapeutic benefits in the adjunctive care of septic patients.

## Introduction

Sepsis is a life-threatening condition caused by a dysregulated immune response to infection, which can lead to multiple organ dysfunction syndrome (MODS). It is one of the leading causes of death in intensive care units globally and remains a major clinical challenge despite advances in antibiotic therapy and supportive care. The pathogenesis of sepsis involves a cascade of inflammatory mediators, reactive oxygen species (ROS), endothelial dysfunction, and subsequent tissue damage [1].

Organ dysfunction in sepsis is often associated with elevated levels of cytokines such as IL-1β, IL-6, and TNF-α and activation of nuclear factor kappa B (NF-κB) pathways. The liver in particular, due to its metabolic and immunologic functions, becomes highly vulnerable to sepsis-induced damage, which was confirmed by increased liver enzyme levels and microscopic findings indicating tissue injury [2].

In parallel, wound healing, a tightly regulated physiological process, is also impaired in septic conditions due to prolonged inflammation, oxidative stress, and immune dysregulation. Sepsis has been shown to impair wound healing by disrupting both the inflammatory and proliferative phases. In a rat model, Rico et al. demonstrated significantly reduced re-epithelialization and collagen synthesis in wounds following sepsis induction [3].

In recent years, attention has turned to natural compounds and dietary phytochemicals as potential adjuncts in the treatment of sepsis. Nayak et al. demonstrated that pomegranate significantly accelerated wound contraction and increased hydroxyproline content in a rat wound model [4]. In the study conducted by Zekavat et al., the application of pomegranate extract was associated with enhanced tissue repair, attributed to its anti-inflammatory and collagen-modulating effects [5].

Recent mechanistic studies have further clarified the beneficial effects of pomegranate extract in the context of sepsis. For example, Trovão et al. demonstrated that pomegranate peel extract prolonged survival in a murine model of lethal sepsis by modulating cytokine release and reducing tissue damage [6]. In another study, Rodrigues et al. reported that punicalagin enhanced the efficacy of meropenem, exerting significant immunomodulatory and antioxidant effects [7].

Therefore, this study aims to evaluate whether pomegranate extract, when administered either before or after sepsis induction, influences wound healing and organ injury in a rat model of cecal ligation and puncture (CLP). It was hypothesized that polyphenols in pomegranate may reduce sepsis-induced tissue damage and enhance healing processes.

## Materials and methods

Animals

Thirty-eight adult male Wistar rats (220-250 g) were obtained from a certified animal care facility at eight weeks of age. All animals were kept under controlled environmental conditions with a 14-hour light/10-hour dark cycle and allowed free access to standard rodent chow and water.

Ethical approval

The study protocol was approved by the Local Ethics Committee for Animal Experiments of Istanbul SBU Mehmet Akif Ersoy Research Hospital (Protocol No. 2018/03).

Study design

Animals were randomly assigned to groups using a computer-generated list and simple randomization. The individual responsible for group assignment was blinded to the treatment protocols to ensure allocation concealment. Rats were divided into four experimental groups. (1) Sham group (n = 8): only laparotomy was performed without CLP or pomegranate extract administration. An oral gavage consisting of 0.5 mL of distilled water was administered daily. (2) Sepsis group (n = 10): CLP surgery was performed to induce polymicrobial sepsis. For seven days, 0.5 mL of distilled water was given by gavage. (3) Pre-sepsis pomegranate group (n = 10): received 100 µL pomegranate extract in 0.5 mL distilled water for seven consecutive days followed by CLP surgery. (4) Post-sepsis pomegranate group (n = 10): Underwent CLP surgery and then received 100 µL of pomegranate extract daily by gavage for seven days. Pomegranate extract (100 µL) obtained by cold pressing of fruit and seed fractions was administered by oral gavage. Although we did not quantify the exact polyphenol or ellagitannin content, the same preparation method and volume were consistently used.

Preparation of pomegranate extract

The pomegranate fruits (*Punica granatum* L.) used in this study were of the Hicaz cultivar and were harvested in October 2023 in Antalya, Turkey. The supplier verified the origin and quality of the fruits, which were stored at 4°C before extract preparation. A total of 3600 g of fresh pomegranate fruits were washed, crushed, and cold pressed. Juice and seed fractions were separated and concentrated under vacuum. The resulting pomegranate extract (combined juice and seed extract) was stored at +4°C until use.

Induction of sepsis

Polymicrobial sepsis was induced using CLP method under sterile conditions [8]. Under anesthesia (ketamine 50 mg/kg and xylazine 10 mg/kg, IP), a midline laparotomy was performed. The cecum was ligated below the ileocecal valve and punctured twice with a 16-gauge needle to induce sepsis. The abdomen was closed with 4-0 prolene sutures.

Sample collection

On postoperative day 7, rats were sacrificed under deep anesthesia. Blood was collected by intracardiac puncture. Tissue samples were obtained from the wound site, liver, kidney, and peritoneum. A portion of each tissue was fixed in 10% formalin for histopathology; the rest was frozen at -80°C. Liver and kidney tissues were stored for simultaneous studies.

Biochemical analysis

Serum samples were analyzed for alanine aminotransferase (ALT), aspartate aminotransferase (AST), creatinine, and urea using standard autoanalyzer techniques.

Histopathologic evaluation

Fixed tissues were processed, embedded in paraffin, sectioned (4 µm), and stained with hematoxylin-eosin. Blind histologic scoring was performed according to the Abramov grading system, which evaluates granulation tissue maturation, lymphocyte infiltration, fibrosis, and acute inflammation on a 0-3 scale [9].

Statistical analysis

Continuous variables were analyzed using the Kruskal-Wallis H test followed by Dunn's post-hoc test with Bonferroni correction. Categorical variables were compared using Fisher's exact test. A p-value of <0.05 was considered statistically significant. Analyses were performed using NCSS 11 and MedCalc version 18.

## Results

Survival and clinical observations

Thirty-eight adult male *Rattus norvegicus* were included in the study. Ten rats in the pre-sepsis pomegranate group were administered 100 μL of pomegranate extract dissolved in 0.5 mL of drinking water via gavage for one week. Animals in the sham group were anesthetized, and their abdomens were opened and closed without any intervention. They then continued their normal lives. The animals in the other group underwent CLP after laparotomy. The animals in the sepsis group were administered 100 μL of pomegranate extract in 0.5 mL of drinking water via gavage daily for one week. They were kept in a 14-hour light/10-hour dark cycle with free access to food and water. A total of 38 animals were initially included in the study. All animals completed the experiment except for those in the pre-sepsis pomegranate group. In this group, due to early clinical deterioration, all animals were sacrificed at an earlier time point for ethical reasons. Additionally, in other groups, some blood samples were insufficient in volume to allow for the full panel of biochemical and cytokine analyses. As a result, sample sizes (n) vary between groups and specific parameters. These discrepancies are clearly indicated in the respective tables. The animals were sacrificed with a high dose of an anesthetic agent after one week. All blood was collected from the body via intracardiac puncture. One animal from the sepsis group was found dead in its cage on the second postoperative day. Animals in the pre-sepsis pomegranate group were euthanized on postoperative day 4 due to deterioration in their general condition. The blood samples obtained from the animals were insufficient for all biochemical tests. The blood volume obtained from 10 animals in the sepsis group was sufficient for cytokine measurement, while only 9 animals had sufficient blood volume for biochemical tests. Blood samples from 8 of the 10 animals in each of the two groups (pre- and post-pomegranate) were sufficient for cytokine measurements, while blood samples from 7 animals were sufficient for biochemical tests. In the sham/control group, blood samples from 7 out of 8 animals were sufficient for cytokine measurements, and blood samples from 6 animals were sufficient for biochemical tests. A summary of group sizes and sample allocation for each assay is presented in Table 1.

**Table 1:** Sample allocation Note: Sample sizes vary due to insufficient blood volume or early sacrifice in the pre-sepsis group. Exact n values are listed for each parameter.

Group	Initial n	Sacrificed day	Biochemistry (n)	Cytokine (n)	Histology (n)
Sham	8	Day 7	6	7	8
Sepsis	10	Day 7	9	10	10
Pre-sepsis pomegranate	10	Day 4	7	7	10
Post-sepsis pomegranate	10	Day 7	7	8	10

Biochemical findings

Urea (mg/dL), creatinine (mg/dL), ALT (U/L), AST (U/L), IL-6 (pg/mL), IL-1 (pg/mL), TNF-α (pg/mL), and presepsin (ng/mL) levels were measured in the blood samples collected. The mean urea level was 39.07 mg/dL in the sham group, 43.25 mg/dL in the sepsis group, 45.57 mg/dL in the group pre-sepsis pomegranate, and 40.29 mg/dL in the group post-sepsis pomegranate (p = 0.581). The mean creatinine level was 0.35 mg/dL in the sham group, 0.33 mg/dL in the sepsis group, 0.31 mg/dL in the pre-sepsis pomegranate group, and 0.25 mg/dL in the post-sepsis pomegranate group (p = 0.013). This difference was statistically significant. The mean AST values were 114.15 U/L in the sham group, 168.38 U/L in the sepsis group, 138.9 U/L in the pre-sepsis pomegranate group, and 137.64 U/L in the post-sepsis pomegranate group (p = 0.61). When comparing ALT values, the value was 51.52 U/L in the sham group, 70.19 U/L in the sepsis group, 36.61 U/L in the pre-sepsis pomegranate group, and 66.87 U/L in the post-sepsis pomegranate group (p = 0.002), and this was statistically significant. When presepsin levels were compared, the values were 4 ng/mL in the sham group, 5.12 ng/mL in the sepsis group, 4.7 ng/mL in the pre-sepsis pomegranate group, and 4.62 ng/mL in the post-sepsis pomegranate group (p = 0.118). IL-6 levels were 1.61 pg/mL in the sham group, 3.51 pg/mL in the sepsis group, 1.83 pg/mL in the pre-sepsis pomegranate group, and 1.63 pg/mL in the post-sepsis pomegranate group (p = 0.180). IL-1 levels were 18.79 pg/mL in the sham group, 13.21 pg/mL in the sepsis group, 9.14 pg/mL in the pre-sepsis pomegranate group, and 11.69 pg/mL in the post-sepsis pomegranate group (p = 0.124). TNF-α levels were 20.52 pg/mL in the sham group, 20.54 pg/mL in the sepsis group, 21.13 pg/mL in the pre-sepsis pomegranate group, and 23.02 pg/mL in the post-sepsis pomegranate group (p = 0.1). Statistically significant differences were only observed in creatinine and ALT levels. These data are detailed in Table 2 and Table 3.

**Table 2:** Biochemical findings (part I) Data are expressed as mean ± standard deviation (SD) and median (min–max) values. Normal reference ranges for each biochemical parameter are also provided. Statistical comparisons among groups were performed using the Kruskal–Wallis test followed by Dunn’s post hoc test. p < 0.05 was considered statistically significant. Sample size varies due to insufficient blood volume in some animals.

Biochemical findings	Reference range		Post-sepsis pomegranate (n = 7)	Pre-sepsis pomegranate (n = 7)	Sepsis (n = 9)	Sham (n = 6)	p-value
Urea	7-20 mg/dL	Mean + SD	40.29 ± 11.78	45.57 ± 10.22	43.25 ± 19.04	39.07 ± 4.71	0.581
		Med (min-max)	40 (26.6-63.1)	46.02 (29.1-55.5)	35.2 (27.3-87)	37.8 (34.5-45)	
Creatinine	0.7-1.3 mg/dL	Mean + SD	0.25 ± 0.06	0.31 ± 0.04	0.33 ± 0.07	0.35 ± 0.03	0.013*
		Med (min-max)	0.22 (0.19-0.34)	0.29 (0.26-0.37)	0.33 (0.27-0.49)	0.36 (0.3-0.4)	
Aspartate aminotransferase (AST)	10-40 IU/L	Mean + SD	137.64 ± 28.49	138.9 ± 43.49	168.38 ± 56.2	114.15 ± 22.76	0.061
		Med (min-max)	134.8 (103-179.9)	136.2 (94.1-227.6)	154.1 (122-306)	102.5 (96.7-148.9)	
Alanine aminotransferase (ALT)	10-40 IU/L	Mean + SD	66.87 ± 13.12	36.61 ± 10.14	70.19 ± 19.98	51.52 ± 8.44	0.002^*^

**Table 3:** Biochemical findings (part II) Data are presented as mean ± standard deviation (SD) and median (min-max) values. Reference ranges for each inflammatory marker are also provided. Groups included in the analysis are sham, sepsis, pre-sepsis pomegranate, and post-sepsis pomegranate. Statistical analysis was performed using the Kruskal-Wallis test followed by Dunn’s post hoc test, as most variables did not follow a normal distribution (based on the Shapiro-Wilk test). p < 0.05 was considered statistically significant. Sample size varies due to insufficient blood volume in some animals. No statistically significant differences were observed among groups for presepsin, IL-6, IL-1, or TNF-α levels (p > 0.05 for all).

Biochemical findings	Reference range		Sepsis (n = 10)	Post-sepsis pomegranate (n = 8)	Pre-sepsis pomegranate (n = 8)	Sham (n = 7)	p-value
Presepsin concentration	12.5-200 pg/mL	Mean + SD	5.12 ± 0.87	4.62 ± 1.11	5.68 ± 2.73	4 ± 0.61	0.118
		Med (min-max)	4.93 (3.86-7.07)	4.9 (3.17-5.92)	4.7 (3.54-11.82)	4.05 (2.9-4.73)	
IL-6 concentration	<2 pg/mL	Mean + SD	3.51 ± 4.05	1.63 ± 0.17	1.83 ± 0.36	1.61 ± 0.12	0.180
		Med (min-max)	1.83 (1.55-13.89)	1.66 (1.33-1.82)	1.78 (1.44-2.61)	1.55 (1.48-1.77)	
IL-1 concentration	<6.5 pg/mL	Mean + SD	13.21 ± 4.61	11.69 ± 3.22	9.14 ± 2.68	18.79 ± 15.84	0.124
		Med (min-max)	11.66 (8.14-24.04)	11.38 (7.67-16.5)	8.83 (5.58-12.89)	12.27 (10.3-53.48)	
TNF-α concentration	<8 pg/mL	Mean + SD	20.54 ± 1.45	23.02 ± 3.09	21.13 ± 1.56	20.52 ± 1.03	0.100
		Med (min-max)	20.14 (18.69-23.23)	22.45 (19.11-29.53)	20.53 (19.19-23.48)	21.11 (18.65-21.4)	

Histopathologic findings

**Figure 1:**
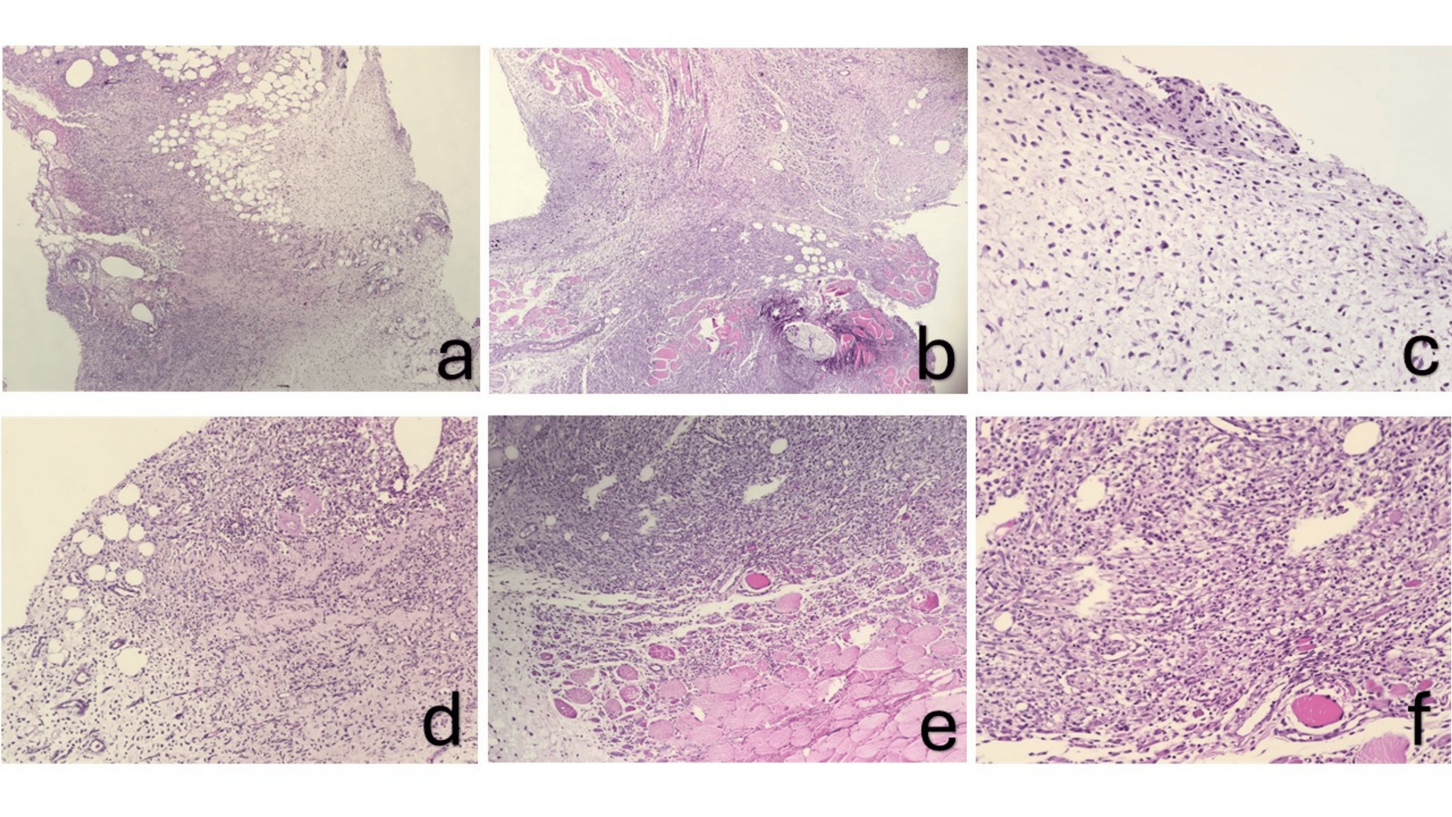
Microscopic images (a) Incision site granulation tissue formation and fibroblastic proliferation area (x10 magnification). (b) Granulation tissue formation in the incision area (maturation 3) (x40 magnification). (c) Fibroblast proliferation and immature collagen accumulation (score 1) (x20 magnification). (d) Lymphocyte infiltration in the inflammation area (score 3) (x20 magnification). (e) Intense lymphocyte infiltration and striated muscle necrosis in the incision area (x10 magnification). (f) Mature granulation tissue formation and striated muscle necrosis in the incision area (x20 magnification).

In histopathological evaluation, significant differences were observed between groups in terms of granulation tissue scores (p = 0.038). In the pre-sepsis pomegranate group, the granulation tissue score was between 0 and 1, which was significantly lower than the scores of 3 and 2-3 in the sepsis and post-sepsis pomegranate groups. This finding suggests that rats in this group exhibited more advanced wound healing. Lymphocyte infiltration scores were similar across all groups. In the sham and sepsis groups, the intensity ranged from 2 to 3, while in the pre-sepsis and post-sepsis pomegranate groups, it was at level 2. This difference was not statistically significant (p = 0.287). Fibrosis findings were similar across all groups, with a score of 1 recorded for each group. No significant difference was detected for this parameter. In terms of acute inflammation, the score was 3 in the sham, sepsis, and pre-sepsis pomegranate groups, while it ranged between 2 and 3 in the post-sepsis pomegranate group. However, this difference did not reach statistical significance (p = 0.067). Granulation tissue was significantly reduced in the pre-sepsis pomegranate group compared to the sepsis and post-sepsis groups, suggesting more advanced healing. However, since the pre-sepsis group was euthanized earlier (day 4), this finding may also reflect insufficient time for tissue proliferation rather than accelerated remodeling. Therefore, these results should be interpreted with caution. Histopathological evaluations are presented in Table 4. Microscopic images of the stages of wound healing are shown in Figure 1.

**Table 4:** Histopathological findings Histological evaluation was performed exclusively on tissue samples obtained from the wound site. Sections were stained with hematoxylin-eosin and evaluated for granulation tissue formation, lymphocyte infiltration, fibrosis, and acute inflammation. Scoring was performed on a scale from 0 (none) to 3 (severe) for each parameter. Results are presented as median scores (with number of animals showing highest score in parentheses when applicable). Statistical comparisons were made using the Kruskal-Wallis test followed by Dunn's post hoc test. p < 0.05 was considered statistically significant. A statistically significant difference was found in granulation tissue scores (p = 0.038), suggesting altered wound healing dynamics between groups.

Parameter	Sham (n = 8)	Sepsis (n = 10)	Pre-sepsis pomegranate (n = 10)	Post-sepsis pomegranate (n = 10)	p-value
Granulation (0-3)	3 (4/8)	3 (9/10)	0–1 (6/10)	2-3 (6/10)	0.038^*^
Lymphocyte infiltration	2-3	2-3	2	2	0.287
Fibrosis	1	1	1	1	Not significant
Acute inflammation	3	3	3	2–3	0.067

## Discussion

The anti-inflammatory and antioxidant properties of the polyphenols found in pomegranate extract are well-known to play an important role in preventing sepsis-induced organ dysfunction and supporting wound healing. Polyphenols exhibit antioxidant effects by scavenging reactive oxygen species (ROS) and reducing oxidative stress [10]. They also limit sepsis-induced organ damage by inhibiting the NF-κB pathway and reducing the levels of proinflammatory cytokines, such as IL-6 and TNF-α. This shortens the inflammatory phase and accelerates the wound healing process. Rasheed et al.'s study supports this mechanism [11].

Our study investigated the effects of using pomegranate extract both before and after sepsis and addressed the question of when to initiate antioxidant therapy in this context. Significantly lower levels of ALT and creatinine in the group that received pomegranate extract before sepsis suggest that initiating antioxidant therapy before sepsis develops may be more effective.

The current literature generally studies the use of pomegranate extract for wound healing topically. For instance, Yuniarti et al. reported that topical pomegranate extract reduced polymorphonuclear leukocyte infiltration [12]. Wang et al. demonstrated that pomegranate extract supports wound healing by utilizing its antimicrobial properties [13].

Similarly, Zhang et al. demonstrated that pomegranate extract is effective in treating burn wounds [14]. Unlike these studies, we investigated the effects of orally administered pomegranate extract on wound healing in the context of sepsis, an important clinical condition in general surgery. The systemic effects observed in our study may be attributable to the absorption and bioactivity of pomegranate-derived polyphenols. Seeram et al. demonstrated that punicalagin and ellagic acid reach measurable plasma levels following oral ingestion in humans [15]. Illescas‑Montes et al. reported in *Nutrients *that punicalagin and ellagic acid enhance human fibroblast proliferation and migration in vitro [16]. Additionally, Giménez‑Bastida et al. showed that urolithin A (a gut-derived ellagitannin metabolite) ameliorates TNF‑α‑induced inflammation in human endothelial cells [17]. These mechanisms may collectively contribute to the observed improvements in both liver injury and wound healing in our study. Furthermore, the presence of less granulation tissue in the group that received pomegranate extract prior to sepsis suggests accelerated progression to the remodeling phase of wound healing. Supporting this, Balachandran et al. emphasized the regulatory role of antioxidants on fibroblast activity and collagen accumulation, which may explain the histological findings observed in our study [18]. In addition, Chen et al., in a systematic review, showed that polyphenols such as ellagitannins exert beneficial effects on systemic inflammation, oxidative stress, and endothelial function [19]. Overall, our findings contribute to the literature by demonstrating that orally administered pomegranate extract may be effective in modulating the sepsis-induced inflammatory response and enhancing wound repair.

While the reduction in granulation tissue could theoretically suggest impaired healing, our histopathological analysis revealed that acute inflammation scores were comparable across all groups. This uniformity in inflammatory response indicates that wound healing was not stifled in the pre-sepsis pomegranate group. Instead, the reduced granulation tissue may reflect a more advanced transition toward the remodeling phase, consistent with the histological patterns observed.

In our study, no significant difference was found between cytokine and AST levels, though a significant decrease in ALT levels was observed. This finding suggests that pomegranate extract may reduce liver damage. This finding is consistent with the literature indicating that pomegranate is effective in modulating inflammatory pathways, such as NF-κB signaling. For instance, Makled et al. reported that pomegranate inhibits TLR4/NF-κB-mediated inflammation and reduces liver enzymes [20]. Similarly, Ali et al. demonstrated that pomegranate extract provides hepatoprotective effects by activating endogenous antioxidant systems [21]. These findings are consistent with the results of our study.

In another study, Aktura et al. reported that kidney damage was reduced in rats that were administered pomegranate extract prior to sepsis [22]. In our study, creatinine levels were significantly lower in the post-sepsis pomegranate group. These studies both indicate the nephroprotective effect of pomegranate.

Another notable finding was that clinical deterioration occurred earlier in the pre-sepsis pomegranate group. This finding is consistent with the results of Tavasoli et al., who reported increased mortality in a CLP-induced sepsis model following pomegranate extract administration [23]. Although we did not assess microbiota composition or bacterial translocation, it is possible that such mechanisms may be involved and warrant further investigation in future studies.

An a priori power analysis was performed based on the expected effect size of the primary outcome, and the number of animals per group was determined accordingly. However, the number of samples analyzed in some cytokine assays was lower (n ≤ 9) due to insufficient blood volume. While the primary endpoints were adequately powered, this may have reduced the sensitivity of the secondary outcomes, particularly when comparing inflammatory markers.

In conclusion, our study provides preliminary evidence that pomegranate extract could reduce multiorgan damage caused by sepsis and encourage wound healing in a rat model. These preclinical findings are consistent with those of previous studies [24,25]. Specifically, Moradnia et al. [24] highlighted the anti-inflammatory and antioxidant potential of *P. granatum* through modulation of oxidative stress pathways, and Sahin et al. [25] demonstrated reduced serum inflammatory cytokines and tissue protection at a histological level in a rat sepsis model. While pomegranate extract is not a standalone treatment, these results suggest that further investigation into its use as a dietary supplement in cases of sepsis is warranted.

However, our study has some limitations. These include a small sample size and the absence of molecular-level investigations, such as those involving NF-κB, iNOS, or oxidative stress markers. Future studies should include longer follow-up periods, dose-response analyses, and detailed molecular profiling.

This study only used male rats. This is because the hormonal variability associated with the estrous cycle in females can affect immune and inflammatory responses. While this increases experimental consistency, excluding female animals is a limitation of the study. There is ample literature documenting gender-based differences in sepsis; therefore, future studies should include both genders [26-28].

In addition, a limitation of the current study is the lack of quantification of the active compounds within the pomegranate extract, such as total polyphenol or ellagitannin concentrations. Although a standardized volume (100 µL) was administered to each animal, the exact dose in mg/kg remains undefined, which may limit reproducibility. Future studies should determine the exact polyphenol content to enhance reproducibility and allow dose-dependent evaluations.

Another limitation is the difference in sacrifice time points between groups. Specifically, the pre-sepsis group was euthanized on day 4 due to early signs of clinical decline, while other groups were evaluated on day 7. This may confound direct comparisons of inflammatory, biochemical, and histopathological findings, as the progression of sepsis and tissue healing could differ between time points. The absence of time-matched control data limits interpretation, and future studies should ensure synchronized sampling intervals to improve validity. This limitation should be considered when interpreting results, as it may artificially suggest accelerated healing or lower inflammation in the pre-sepsis group, which could instead reflect the earlier time point rather than the treatment effect.

In summary, the main limitations of this study are the small sample size, the inconsistent timing of tissue harvesting due to early deaths, and the absence of mechanistic evaluations at the molecular level. These factors should be taken into account when interpreting the results and designing future studies.

## Conclusions

Systemic administration of pomegranate extract prior to sepsis induction improves wound healing and reduces liver injury in a rat model of polymicrobial sepsis. These results suggest that pomegranate may be a promising adjunctive therapeutic agent in sepsis management. However, further mechanistic studies and clinical trials are required to confirm its use in human subjects.
